# Resting-state alpha power is selectively associated with autistic traits reflecting behavioral rigidity

**DOI:** 10.1038/s41598-018-30445-2

**Published:** 2018-08-10

**Authors:** Virginia Carter Leno, Samuel B. Tomlinson, Shou-An A. Chang, Adam J. Naples, James C. McPartland

**Affiliations:** 1 Yale Child Study Center, 230 South Frontage Road, New Haven, 06520 CT USA; 2 Institute of Psychiatry, Psychology & Neuroscience, King’s College London, 16 De Crespigny Park, London, SE5 8AF UK; 3 School of Medicine and Dentistry, University of Rochester Medical Center, Rochester, 14642 NY USA

## Abstract

Previous research suggests that variation in at-rest neural activity correlates with specific domains of the ASD phenotype; however, few studies have linked patterns of brain activity with autistic trait expression in typically developing populations. The purpose of this study was to examine associations between resting-state electroencephalography (EEG) and three domains of the broader autism phenotype (social interest, rigidity, and pragmatic language) in typically developing individuals. High-density scalp EEG was recorded in thirty-seven typically developing adult participants (13 male, aged 18–52 years). The Broad Autism Phenotype Questionnaire (BAP-Q) was used to measure autistic trait expression. Absolute alpha power (8–13 Hz) was extracted from eyes-closed epochs using spectral decomposition techniques. Analyses revealed a specific positive association between scores on the BAP-Q Rigidity subscale and alpha power in the parietal scalp region. No significant associations were found between alpha power and the BAP-Q Aloofness or Pragmatic Language subscales. Furthermore, the association between EEG power and behavioral rigidity was specific to the alpha frequency band. This study demonstrates that specific traits within the broader autism phenotype are associated with dissociable patterns of at-rest neural activity.

## Introduction

Autism spectrum disorder (ASD) is a neurodevelopmental disorder characterized by persistent impairments in social communication, restrictive, repetitive behaviors, and atypical response to sensory information^1^. Given its early developmental course^2^ and high heritability both for diagnostic status and continuous phenotypes^3,4^, ASD is posited to be neurobiological in origin; however, the exact etiologies of ASD remain unclear. While ASD is recognized as a single clinical entity, tremendous heterogeneity exists across individuals receiving the diagnosis, and previous research suggests that the different symptom domains of ASD may represent dissociable (or at least partially-dissociable) disorder states altogether^5^. Examining symptom domains independently may reveal specific patterns of neural activity associated with the different clinical dimensions of the ASD phenotype, and illuminate underpinning neurobiological mechanisms.

Scalp electroencephalography (EEG) is frequently used to compare patterns of brain activity between individuals with ASD and typically developing counterparts. Although much research has focused on electrical responses to specific neurocognitive tasks (i.e., ‘task-dependent’ or ‘event-related’ activity), researchers have increasingly attributed significance to brain activity recorded in the absence of task demands (i.e., ‘task-free’ or ‘resting-state’ EEG)^6^. Among the most consistent findings observed across resting-state studies in ASD are alterations in power in the alpha frequency range (8–13 Hz). Widespread increases in resting-state alpha power have been described in both children^7,8^ and adults with ASD^9^ compared to typically developing individuals, although other studies have reported contradictory findings^10–12^. Relatively few studies have explored how resting-state alpha power correlates with specific domains of the ASD phenotype. One study found that children with ASD who displayed greater right frontal alpha asymmetry (i.e., increased alpha power in the right hemisphere compared to the left hemisphere) had higher levels of social impairment and better visual analytic skills than those with left frontal asymmetry^13^. Dawson and colleagues^11^ found that children classified as socially ‘passive’ exhibited reduced frontal alpha power compared to those who were ‘active-but-odd.’ Another study identified a link between specific ASD symptoms (e.g., extreme attention to detail) and variations in alpha power in the parietal lobe^9^. Thus, existing literature suggests that oscillatory activity within the alpha frequency range may be independently associated with specific domains of the ASD phenotype.

Some researchers argue that ASD represents the upper extreme of a continuous spectrum of traits observed throughout the general population^14^. Accordingly, comparable patterns of neural activity have been described in individuals with ASD and typically developing individuals with high levels of autistic traits, suggesting that neural alterations associated with ASD may also be present in the subclinical population^15,16^. To date, however, few studies have explored how resting-state EEG activity relates to autistic trait expression in typically developing populations. One EEG study identified an inverse relationship between autistic trait expression, as measured by the Social Responsiveness Scale (SRS^17^), and functional connectivity strength, particularly in the lower frequency (<10 Hz) range^17^. A more recent study found that higher levels of autistic traits were associated with increased relative alpha power^18^, and this effect appeared to be driven by the ‘Aloofness’ symptom domain as measured by the Broad Autism Phenotype Questionnaire (BAP-Q^20^). This limited literature suggests that specific associations between domains of the ASD phenotype and resting-state EEG activity may extend to subclinical populations.

The current study examined associations between resting-state EEG alpha power and specific domains of the broader autism phenotype within a sample of typically developing adults, thereby extending previous work, which has mostly focused on associations with overall ASD symptom expression. We utilized the BAP-Q to measure three independent subscales (Aloofness, Rigidity, Pragmatic Language) thought to index three symptom domains delineated within the Diagnostic and Statistical Manual of Mental Disorders (DSM-IV): social withdrawal, restricted and repetitive behavior and interests, and impairments in communication, respectively. We also build on more recent studies (e.g.^19^), by recruiting a larger sample size with a wider age range and using a denser electrode array for EEG data collection. We hypothesized that there would be dissociable correlations between resting-state alpha power and specific domains of traits encompassed by the broader autism phenotype.

## Method

### Participants

Typically developing adults were recruited locally (New Haven, Connecticut) through flyers and word-of-mouth advertising. Participants were screened for the following inclusion criteria:(1) reported age ≥18 years; (2) no reported history of chronic illness, psychiatric diagnoses (including ASD), or seizures; (3) no ongoing heart conditions; (4) no ongoing use of medications known to impact the EEG. Individuals with history of motor impairments, recent brain injury, and/or congenital brain malformation were ineligible for the study. All participants reported normal or corrected-to-normal vision and hearing. Of the 41 recruited participants, four were excluded prior to analysis (EEG data corruption, n = 3; post-testing report of psychiatric diagnosis, n = 1) to yield a final sample of 37 participants (13 male, 24 female) with a mean age of 23 years (range = 18.5–52.7). The majority of participants (89.2%) reported right-hand dominance. See Table 1 for additional demographic information. This study was approved by the Yale University Institutional Review Board (IRB) and was performed in accordance with relevant guidelines and regulations. Informed consent was obtained from all participants. Participants were compensated $40 for their time.

**Table 1:** Sample Demographics and Scores on the Broad Autism Phenotype Questionnaire (BAP-Q).

Age (SD, range)	23.0 years (6.3; 18.5–52.7)
Gender (M: F)	13 Male: 24 Female
Ethnicity, n, (%)	
Caucasian	18 (48.6)
Asian	7 (18.9)
African American	4 (10.8)
Hispanic	2 (5.4)
Black/West Indian	2 (5.4)
Other	4 (10.8)
BAP-Q Subscales (SD; range)	
Aloofness	32.2 (9.9; 16–54)
Pragmatic Language	29.8 (7.9; 12–46)
Rigidity	34.2 (10; 13–53)

### Measures

#### Questionnaires

Participants completed the Broad Autism Phenotype Questionnaire (BAP-Q)^20^. The BAP-Q is a 36-item self-report instrument designed to measure aspects of the ASD phenotype in typically developing individuals. Previous studies suggest that the BAP-Q is the most appropriate instrument for measuring autistic traits in the typically developing population^21^. Individual items are scored using a 6-point Likert scale (1 = ‘very rarely applies,’ 6 = ‘applies very often’) and grouped according to three subscales: Aloofness, Rigidity, and Pragmatic Language. The Aloofness subscale measures lack of interest in or enjoyment of social interaction; the Rigidity subscale indexes difficulty adjusting to change and a tendency towards repetitive behaviors; and the Pragmatic Language subscale indexes difficulties in the social aspects of language and communication^19^. The three subscales are designed to map onto the three DSM-IV symptom domains of ASD^22^: social deficits, stereotyped and repetitive behaviors, and social language deficits, respectively. Prior work finds the internal consistency of the BAP-Q subscales to be good to excellent, with Cronbach’s alpha, α = 0.94 for the Aloof subscale, α = 0.85 for the Pragmatic subscale, α = 0.91 for the Rigidity subscale^20^. Internal consistency in our sample was good for the Aloofness and Rigidity subscales (α = 0.88 and 0.85, respectively) and acceptable for the Pragmatic Language subscale (α = 0.72). See Table 1 for sample mean scores across subscales.

#### EEG Protocols

High-density scalp EEG was recorded using a 128-channel HydroCel Geodesic Sensor Net system (Electrical Geodesics, Eugene, OR) with 500 Hz sampling frequency. Voltages were referenced online to the vertex electrode (Cz) and re-referenced offline to the average reference. Impedances were maintained below 40 kΩ prior to each recording block. For each participant, approximately six minutes of resting-state EEG activity (~2 mins Eyes-Closed, ~4 mins Eyes-Open) were collected. Verbal cues delivered through E-Prime experimental design software (Psychology Software Tools, Pittsburg, PA) instructed participants to remain still and relaxed with eyes opened or closed. Resting-state recordings were acquired as part of a larger study of reward processing (see^23^ for additional details regarding EEG acquisition).

Segments of resting-state EEG were divided into non-overlapping 2000 millisecond (ms) epochs and grouped by condition (i.e., ‘Eyes-Open’ and ‘Eyes-Closed’). Epochs were visually inspected by two authors (SBT; SAC) using EEGLAB v13.5 software^24^. Single epochs containing eye blinks, vertical or horizontal eye movements, motor artifact, and/or excessive EEG irregularities were manually eliminated. Four channels positioned anteriorly to record electrooculographic activity were excluded. Due to extensive artifact in the ‘Eyes-Open’ condition leading to a large percentage of trials rejected, only epochs from the ‘Eyes-Closed’ condition were analyzed.

#### Spectral analysis

Approximately two minutes of resting-state EEG activity were collected, epoched, and visually inspected for each participant. On average, 27.2 epochs (minimum = 15, maximum = 32) were analyzed per participant after manual epoch rejection. Epochs were linearly de-trended, zero-padded, and smoothed using a 1 Hz taper. Spectral decomposition was performed using the multi-taper method implemented in the *ft_freqanalysis* function of the FieldTrip analysis package^25^. For each participant, the mean absolute alpha power over epochs was computed at each electrode by averaging power spectra (μV^2^/Hz) across the 8–13 Hz frequency range. Regional analysis of alpha power was performed by clustering electrodes into distinct regions of interest (ROI), using the International 10/20 Electrode Placement System as a guide. Previous reviews have described high levels of resting-state alpha activity in the midline regions^26^. Therefore, three midline ROIs were designated around electrodes Fz (frontal: 4, 5, 10, 11 (Fz), 12, 15, 16, 18, 19), Cz (central: 6, 7, 13, 30, 31, 37, 80, 87, 105, 106, 112), and Pz (parietal: 52 (P3), 53, 54, 55, 60, 61, 62 (Pz), 67, 72, 77, 78, 79, 85, 86, 92 (P4)).

#### Statistical analysis

All analyses were completed in Stata 14. Where relevant, all variables were checked for normality by inspection of histogram plots. To identify the scalp regions with maximal alpha power, average alpha power within each ROI (Fz, Cz and Pz) was compared via one-way repeated measures analysis of variance (ANOVA) followed by post-hoc contrasts using Tukey Honestly Significant Difference (HSD). Computation of associations between alpha power and BAP-Q scores was focused on the scalp region displaying maximal power, applying two approaches. First, an electrode-level analysis was performed by computing the Pearson correlation (*r*) between alpha power and the three BAP-Q subscales (Aloofness, Rigidity, Pragmatic Language) at each electrode within the designated ROI. Planned t-tests were used to compare the average of electrode-level correlations between alpha power and each of the three BAP-Q subscales. Second, a participant-level analysis used multiple regression to test for an association between the three BAP-Q subscales (entered as separate predictor variables) and mean alpha power across the designated ROI (entered as the dependent variable). Collinearity statistics indicated that despite significant correlations between the subscales *(rs* = 0.36–0.47), entering the three subscales simultaneously as predictor variables was acceptable (each variance inflation factor (VIF) <2). Where trend or significant associations were found, results were adjusted for age and sex, then adjusted for the individual number of analyzed epochs by including these variables as regression co-variates.

To test whether any relationships were specific to the alpha frequency range, we simultaneously estimated the individual associations between BAP-Q Rigidity and power in five frequency bands (delta, theta, alpha, beta, gamma) using multivariate regression, and the strength of the coefficient in the BAP-Q Rigidity-alpha power association within the specified ROI was compared against all other coefficients using post-estimation tests. Here, BAP-Q Rigidity scores were entered as the predictor variable, and power in the five frequency bands as dependent variables. All tests were two-tailed and alpha was set at 0.05.

#### Data availability

The datasets generated during and/or analyzed during the current study are available from the corresponding author upon reasonable request.

## Results

### Maximal Alpha Power Across the Scalp

**Figure 1:**
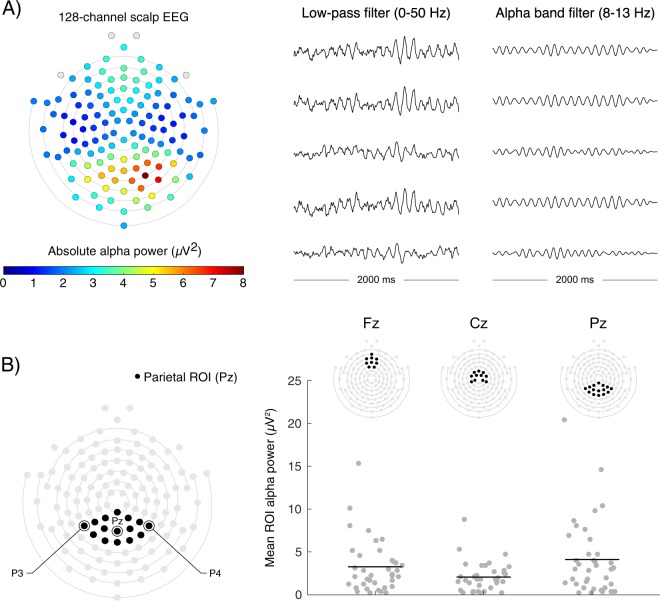
(**A**) Scalp-wide distribution of absolute alpha power. (**B**) Comparison of absolute alpha power across the frontal (Fz), central (Cz) and parietal (Pz) ROIs.

The repeated measures ANOVA revealed significant differences in mean alpha power across ROIs (F(2, 72) = 14.84, *p* < 0.01; Fig. 1), and post-hoc contrasts revealed greater mean alpha activity in the parietal ROI (mean alpha activity = 4.11 μV^2^, *SD* = 4.35) relative to the frontal (mean alpha activity = 3.20 μV^2^, *SD* = 3.10; post-hoc contrast *p* < 0.05) and central ROIs (mean alpha activity = 2.06 μV^2^, *SD* = 1.73; post-hoc contrast *p* < 0.01). Therefore, analysis of alpha activity and phenotypic characteristics focused on the parietal ROI.

### Association between Alpha Power and Individual BAP-Q Subscales

**Figure 2:**
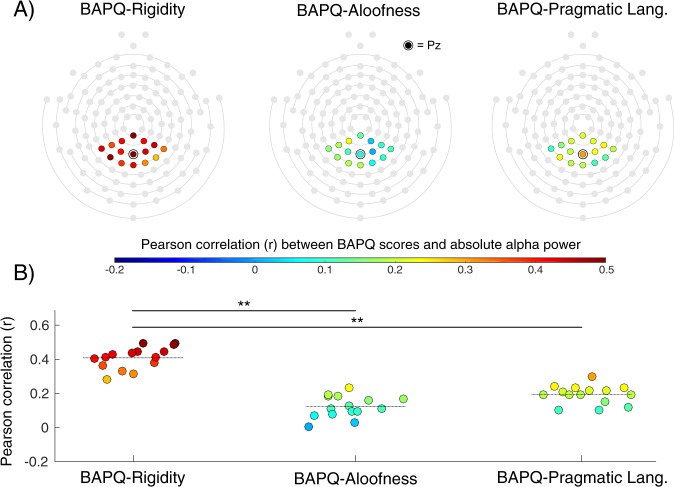
(**A**) Correlations between BAP-Q subscale scores and absolute alpha power at each electrode in the parietal ROI. (**B**) Visual comparison of the correlation between each BAP-Q subscale (Rigidity, Aloofness, and Pragmatic Language) and alpha power at individual electrodes within the parietal ROI. Dotted lines represent the average correlation across the ROI. **p < 0.01.

Across electrodes in the parietal ROI, the mean correlation between alpha power and BAPQ-Rigidity (*r* = 0.41, *SD* = 0.06) significantly exceeded the average correlation observed for the Aloofness (*r* = 0.12, *SD* = 0.06; contrast *p* < 0.01) and Pragmatic Language (*r* = 0.19, *SD* = 0.05; contrast *p* < 0.01) subscales (Fig. 2).

**Figure 3:**
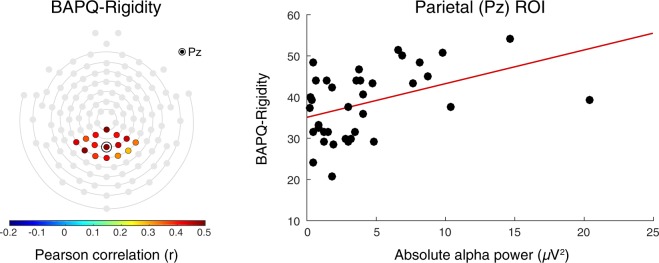
*Left*: Heat map depicting the correlation between absolute alpha power and BAP-Q Rigidity scores at each electrode in the parietal ROI. *Right*: Association between mean absolute alpha power across the parietal ROI and BAP-Q Rigidity scores.

Across participants, multiple regression revealed a significant, positive association between BAPQ-Rigidity scores and mean alpha power across the parietal ROI (β = 0.20, SE = 0.08, *p* < 0.05) (Fig. 3). No significant associations were found between mean alpha power and the Aloofness (β = −0.05, SE = 0.08, *p* = 0.52) or Pragmatic Language (β = 0.05, SE = 0.10, *p* = 0.63) subscales. The association between BAPQ-Rigidity scores and alpha power remained significant after adjusting for age and sex (β = 0.21, SE = 0.08, *p* < 0.05) and after adjusting for the number of available epochs for analysis (β = 0.18, SE = 0.08, *p* < 0.05).

### Specificity of Association between Alpha

#### Power and BAP-Q Rigidity

After identifying a significant relationship between alpha power in the parietal ROI and BAP-Q Rigidity scores, we next examined whether the association was *specific* to this frequency range. Results showed that only power in the alpha frequency range was significantly associated with BAP-Q Rigidity, as associations between BAP-Q Rigidity and all other frequency bands (delta, theta, beta, gamma) were non-significant (*ps* ranged from 0.08 (beta frequency range) - 0.35 (gamma frequency band)). This pattern of results remained in co-variation analyses. Post-estimation tests (adjusting for age and sex) showed that the alpha power - BAP-Q Rigidity association was significantly different from the power - BAP-Q Rigidity association in all other frequency bands (all *ps* < 0.05) aside from the delta frequency band (*p* = 0.18).

## Discussion

Previous research has revealed differences in at-rest brain activity between individuals with clinically-diagnosed ASD and their typically developing counterparts. However, few studies to date have examined resting-state EEG activity in samples of typically developing individuals with varying degrees of subthreshold ASD trait expression. In the present study, we recorded high-density, resting-state scalp EEG and examined correlations between band-specific power and scores on three subscales of the BAP-Q instrument. Primary analyses revealed a positive association between resting-state alpha power (8–13 Hz) and scores on the BAP-Q Rigidity subscale in the parietal region. This association was domain-specific, as no significant associations were identified between alpha power and the other two BAP-Q subscales. Furthermore, follow-up analyses found no significant association between the Rigidity subscale and oscillatory power in the other frequency bands.

Previous studies of resting-state brain activity have identified associations between alpha power and global measures of the ASD phenotype (i.e., without examining specific domains of function). Both EEG and magnetoencephalography (MEG) studies have found increased alpha power among individuals with ASD compared to typically developing peers, particularly in posterior and centro-parietal regions^7,8^. Critically, these studies found that the magnitude of alpha power correlated with *overall* ASD symptom severity (although it should be noted that a limited number of opposing findings have also been reported, see^10,12^). Our study builds upon this literature by suggesting that the correlation between alpha power and global ASD symptom severity may be driven by specific subdomains of the broader autism phenotype, namely, the restricted and repetitive behaviors domain. Indeed, it is possible that this specific domain of the ASD phenotype may have driven the association between alpha power and global ASD severity reported in the aforementioned studies (e.g.^7,8^). Future research should therefore investigate not only associations between neural functioning and overall severity, but also with specific symptom domains.

Currently, research examining specific domains of the broader autism phenotype is limited despite evidence that these domains may have dissociable neurobiological and genetic correlates^5^. Taking this sub-domain approach may help to explain the heterogeneity within ASD from a neurobiological point of view and is in keeping with recent calls for research focusing on the neural correlates of different aspects cognition and behavior, rather than diagnostic status^27^. In terms of previous literature, one EEG-based study of individuals with ASD found that increased resting-state alpha power in the posterior scalp region correlated with increased ‘attentiveness to detail’^9^. The authors found no evidence for an association between alpha power and other domains of the ASD phenotype, suggesting that the relationship was symptom-specific. Critically, although several studies have now linked resting-state alpha power to specific domains of the ASD phenotype, the functional significance of resting-state EEG oscillations is incompletely understood^6,28^, and furthermore, evidence suggests that the correlates of oscillatory information may differ between ASD and typically developing populations^29^. Evidence from event-related (i.e., task-dependent) EEG studies suggests that alpha oscillations are involved in suppressing neural responses^30,31^. Similarly, resting-state alpha activity may be required to attenuate the brain’s response to sensory input in the absence of task demands. Participants with decreased resting-state alpha activity may therefore exhibit increased sensitivity to environmental novelty, which may favor an aversion to change and a preference for repetitive, stereotyped routines. However, this interpretation is speculative and requires investigation in future research.

To our knowledge, only one prior study has examined the association between resting-state EEG power and different domains of autistic trait expression among typically developing individuals. In contrast to current results, the previous study found that increased alpha power in the central and parietal scalp regions correlated with increased scores on the BAP-Q Aloofness subscale^18^. Differences in how EEG data was collected, and how alpha power was measured could have contributed to the discrepancies in results. The previous study measured alpha activity by computing the difference between power in the eyes-closed versus eyes-open conditions, whereas the current study only examined alpha power during the eyes-closed condition due to the presence of extensive artifact in the eyes-open condition. This should be held in mind when interpreting current results, as previous work suggests there may be differences in the strength and topography of oscillatory activity between eyes-open and eyes-closed paradigms^32^. We posit that measuring a difference score between eyes-open and eyes-closed alpha activity (as was done in^19^) may index a fundamentally distinct neurophysiological process than measuring eyes-closed activity independently. Furthermore, the interpretation of the prior study focused primarily on alpha activity in more lateral parietal regions, more consistent with the distribution of ‘mu’ activity^19^, whereas our analysis highlighted the centro-parietal region. Nevertheless, future work is needed to reconcile these results and further investigate the manner in which specific computations of alpha power (e.g., eyes open vs. eyes closed vs. differential) may index distinct functional processes.

Several limitations of the present design are being addressed in ongoing research by our group. First, the study included a modest sample size and thus requires replication. Our constrained sample size may have limited our ability to detect smaller associations among alpha power and the other subscales or between the Rigidity subscale and the other frequency bands. Additionally, as we did not have any information regarding the cognitive ability of participants, we could not control for intellectual ability in our analyses. Notably, others have found no association between intellectual ability and resting-state alpha power in typically developing individuals^19^. Similarly, one study found an association between cognitive function and alpha power in ASD, but no such association was observed in the typically developing comparison group^23^. However, the present study cannot rule out this potential effect. The main strengths of the current work are the novel approach to understanding brain-behavior associations relevant to the ASD phenotype, and the in-depth exploration of the specificity of said associations. If the current pattern of results were replicated in clinical samples, this would suggest that certain metrics of neural functioning, such as resting-state EEG power, could be used in conjunction with behavioral assessments to identify in which particular domains of functioning (e.g. social motivation, restricted and repetitive behaviors, flexible and effective communication) an individual may require greatest support.

To conclude, the present results align with the emerging notion that specific domains of the ASD phenotype exhibit partially-dissociable electrographic correlates^5^, even in the typically developing (i.e., subclinical) population. Additional work is needed to understand the functional significance of resting-state oscillations and their contributions to heterogeneity within the broader autism phenotype.
